# Padel tennis: Spectrum of injuries and the role of radiological imaging

**DOI:** 10.4102/sajr.v30i1.3506

**Published:** 2026-10-08

**Authors:** Kapil Shirodkar, Iqbal Siddi Ganie, Sonal Saran, Rahij Anwar, Kartikeyan P. Iyengar, Rajesh Botchu, Shalendra K. Misser

**Affiliations:** 1 Department of Musculoskeletal Radiology, Faculty of Radiology, Royal Orthopaedic Hospital, Birmingham, United Kingdom; 2 Department of Musculoskeletal Radiology, Faculty of Radiology, Lakesmit and Partners Inc, Durban, South Africa; 3 Department of Radiology, All India Institute of Medical Sciences, Rishikesh, India; 4 Department of Orthopaedics, Medway and Maritime Hospital, Gillingham, United Kingdom; 5 Department of Orthopaedics, Southport and Ormskirk Hospital, Southport, United Kingdom; 6 West Lancashire Hospital National Health Service Trust, Southport, United Kingdom; 7 Department of Musculoskeletal Radiology, Royal Orthopaedic Hospital, Birmingham, United Kingdom; 8 Department of Radiology, Lakesmit and Partners Inc, Durban, South Africa

**Keywords:** padel tennis, sports injuries, athletes, racquet sports, sports trauma, CT, MRI, ultrasound

## Abstract

Padel, a fast-paced, high-intensity racquet sport, is associated with a wide range of musculoskeletal injuries. These injuries can range from acute traumatic events, such as ligament tears and fractures, to chronic overuse syndromes, such as tendinitis and stress fractures. The use of imaging plays a crucial role in the diagnosis and management of these conditions. A thorough understanding of the sport’s biomechanics and injury patterns is essential for prompt and targeted intervention. While plain radiographs are useful for identifying fractures, ultrasonography is particularly effective for visualising soft tissue injuries. CT can provide detailed insight into complex fractures, while MRI excels in the evaluation of subtle bone and soft tissue injuries that may not be visible on other imaging modalities. This article reviews common padel-related injuries, their biomechanics and the roles of various imaging methods in their assessment.

## Introduction

Padel, the in-vogue racquet sport on an exponential rise, is capturing professional and recreational players’ hearts.^1,2^ This doubles sport, first played in Acapulco, Mexico, in 1962,^1^ requires a smaller court with enclosed walls, combining the finest elements of squash and tennis. The unique playing conditions result in a fast-paced, physically demanding sport that requires high body endurance and agility for quick changes of directions and movements, leading to some characteristic injury patterns. Radiologists must know these injury patterns, as they will be encountered on imaging and play a significant role in the diagnosis of padel-related injuries, helping guide appropriate management and aiding timely recovery. This pictorial review is based on a retrospective analysis of imaging data and clinical records from padel players presenting to our sports imaging and musculoskeletal radiology department over a 2-year period, supplemented by a systematic review of the current literature on padel-related injuries.

## Epidemiology and incidence

The injury rate is approximately three per 1000 training hours and eight per 1000 matches.^3,4^ The elbow is the most frequently injured body part, followed by the lower back, knee and shoulder, with common extensor tendinopathy, also known as tennis elbow, being the most commonly reported pathology.^5^ In terms of tissue type, muscles are the most affected, followed by ligaments.^6^ Elite athletes tend to have a higher incidence of injury and more significant muscle injuries, while amateur players sustain more severe injuries and a greater proportion of tendon injuries. Male players experience more injuries than their female counterparts, particularly with respect to muscle and ligament damage in the shoulder and tendinopathies around the elbow. In contrast, women are more susceptible to muscle injuries in the shoulder and arm, ligament injuries around the elbow and bone injuries in the wrist and elbow.^7^ Non-contact is the most prevalent mechanism of injury, with injuries often occurring towards the end of matches. The selection of strokes also varies with the level of player experience, with beginners tending to use more groundstrokes and lobs, whereas professional players focus on volleys, wall strokes and smashes.^8,9^

Several intrinsic and extrinsic risk factors also contribute to injury. Intrinsic factors include older age (> 35 years), high BMI and prior joint problems.^10,11^ Extrinsic factors are modifiable, including poor technique, inadequate warm-up and equipment (e.g. grip size or string tension).

## Padel: Anatomy and biomechanics

Padel biomechanics involves analysing the racquet swing, footwork and body posture.^12^ Repetitive, high-velocity motions and abrupt directional changes on a compact court, place immense stress on the musculoskeletal structures. During serves and overheads, rapid shoulder rotation and elbow extension can generate ball speeds that exceed 150 km/hr.^9^ The small court size forces players to make sudden pivots and lunges, which transmit considerable torsional and compressive loads through the hips, knees and ankles. These demanding biomechanics lead to both acute trauma and overuse injuries. The presence of glass walls also presents a unique hazard for impact injuries that are not seen in other racquet sports. Understanding these biomechanics provides a contextual framework for the radiologist’s search pattern when faced with a potentially injured athlete.

## Upper extremity injuries

### Shoulder injuries

The shoulder’s mobility facilitates powerful serves, but its instability makes it prone to injury. Repetitive overhead movements, such as serving and smashing, require a combination of fast shoulder and elbow rotations, along with hyperextension of the back,^12,13^ which induces the most common shoulder injuries: rotator cuff tears, tendinitis and impingement.^12^ The dominant shoulder typically bears the brunt of these forces, especially in competitive players or those with improper technique. Hitting the ball with one leg positioned behind the other significantly increases the stress on the shoulder muscles and tendons.^14^ Plain radiographs are the first-line imaging modality in acute shoulder trauma, demonstrating fractures such as the comminuted proximal humeral fracture displayed in Figure 1.

**FIGURE 1:**
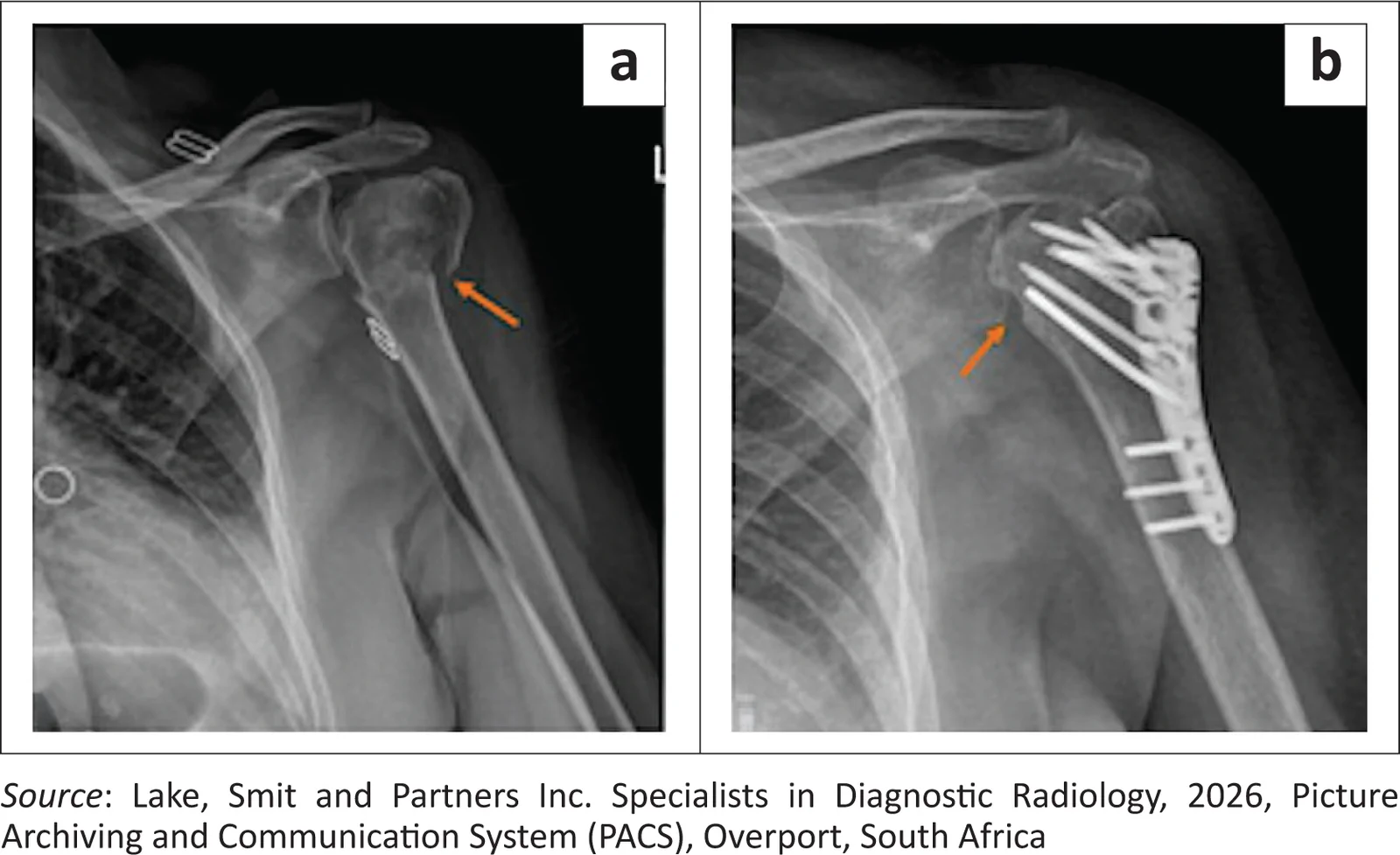
Fracture of the neck and head of the humerus of a 50-year-old female padel player who presented with acute onset left shoulder pain after a fall on the court: (a) frontal radiograph revealed a comminuted fracture of the head and neck of the left humerus (arrow) and (b) post-operative radiograph showing plate and screw fixation of the left humeral fracture (arrow).

MRI is the modality of choice for evaluating shoulder pain in padel athletes, with the supraspinatus tendon most frequently torn.^15^ MRI can accurately assess the extent of tendon damage and detect any concomitant muscle atrophy or retraction. Labral tears, which are a common source of shoulder discomfort and instability, resulting from repetitive overhead motion or acute trauma, are best visualised with MR arthrography or high-field standard MRI.^16,17^ Other shoulder injuries that can be seen in padel players include impingement syndrome, biceps tendinopathy and acromioclavicular joint disorders, all of which can be distinguished by MRI to guide appropriate treatment.

The size and configuration of a tear determine the management approach; small partial-thickness tears may be treated conservatively with rest, physical therapy and injections, while full-thickness tears often require surgical repair. Advanced imaging plays a crucial role in guiding this decision-making process, and post-operative MRI can be used to evaluate the healing response.^12,13,14^ MRI is the preferred modality for characterising labral pathology, distinguishing true anterior labral tears from normal labral variants and recesses, as illustrated in Figure 2.

**FIGURE 2:**
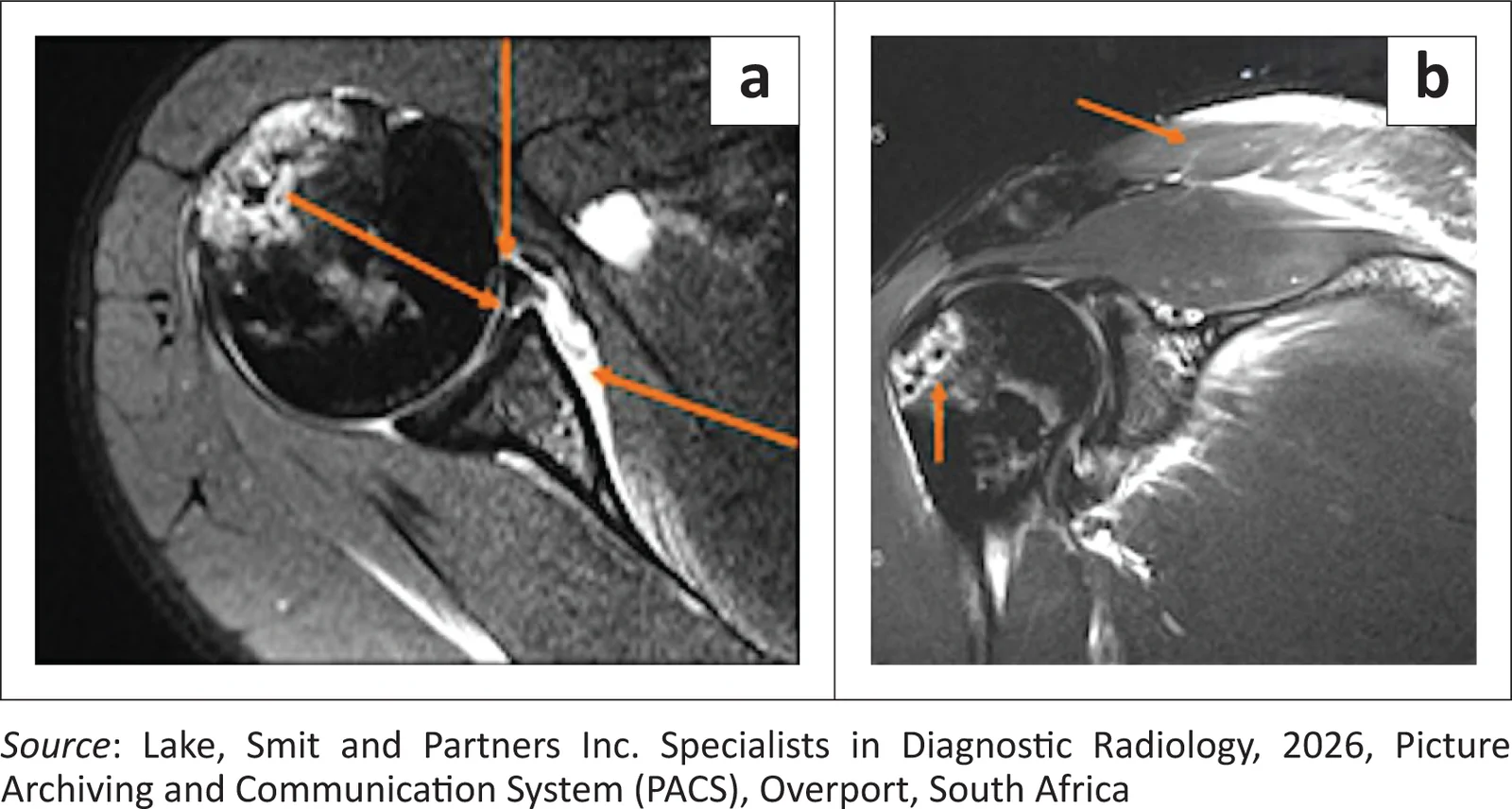
(a) Axial proton density fat saturated (PDFS) image of the right shoulder demonstrating injury to the glenoid articular cartilage, anterior labrum and anterior glenoid periosteum (arrows) and (b) coronal PDFS image of the right shoulder showing deltoid muscle and right humerus bony contusion (arrows).

### Elbow injuries

The elbow is a commonly injured joint in padel, with lateral epicondylitis (tennis elbow) and medial epicondylitis (golfer’s elbow) being frequently seen due to the repetitive movements of the arm.^1,13,18^ Lateral elbow tendinosis develops from repeated ball striking and rapid grip changes, involving micro-tears in the forearm extensor tendons at the lateral epicondyle.^18^ While chronic cases may show calcification on plain radiographs, MRI and ultrasonography (USG) are the modalities of choice to assess the severity of the tendinopathy and direct treatment.^19^ (Figure 3 and Figure 4).

**FIGURE 3:**
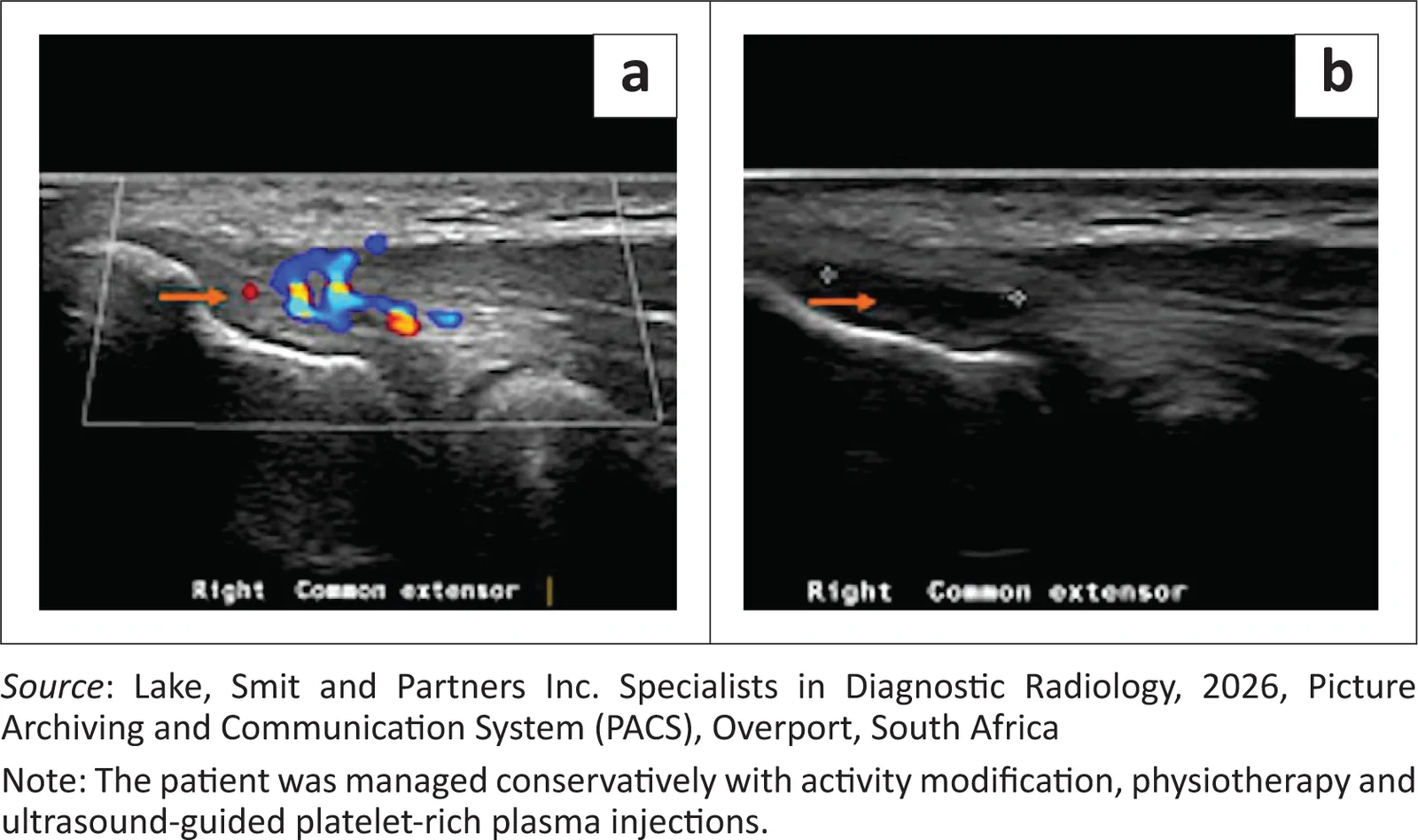
Lateral epicondylitis of a 44-year-old male player with persistent lateral elbow pain. Ultrasound demonstrated thickening of the common extensor tendon with: (a) hypoechoic areas and increased vascularity on Doppler imaging and (b) features consistent with lateral epicondylitis with a focal partial-thickness intrasubstance tear within the common extensor tendon.

**FIGURE 4:**
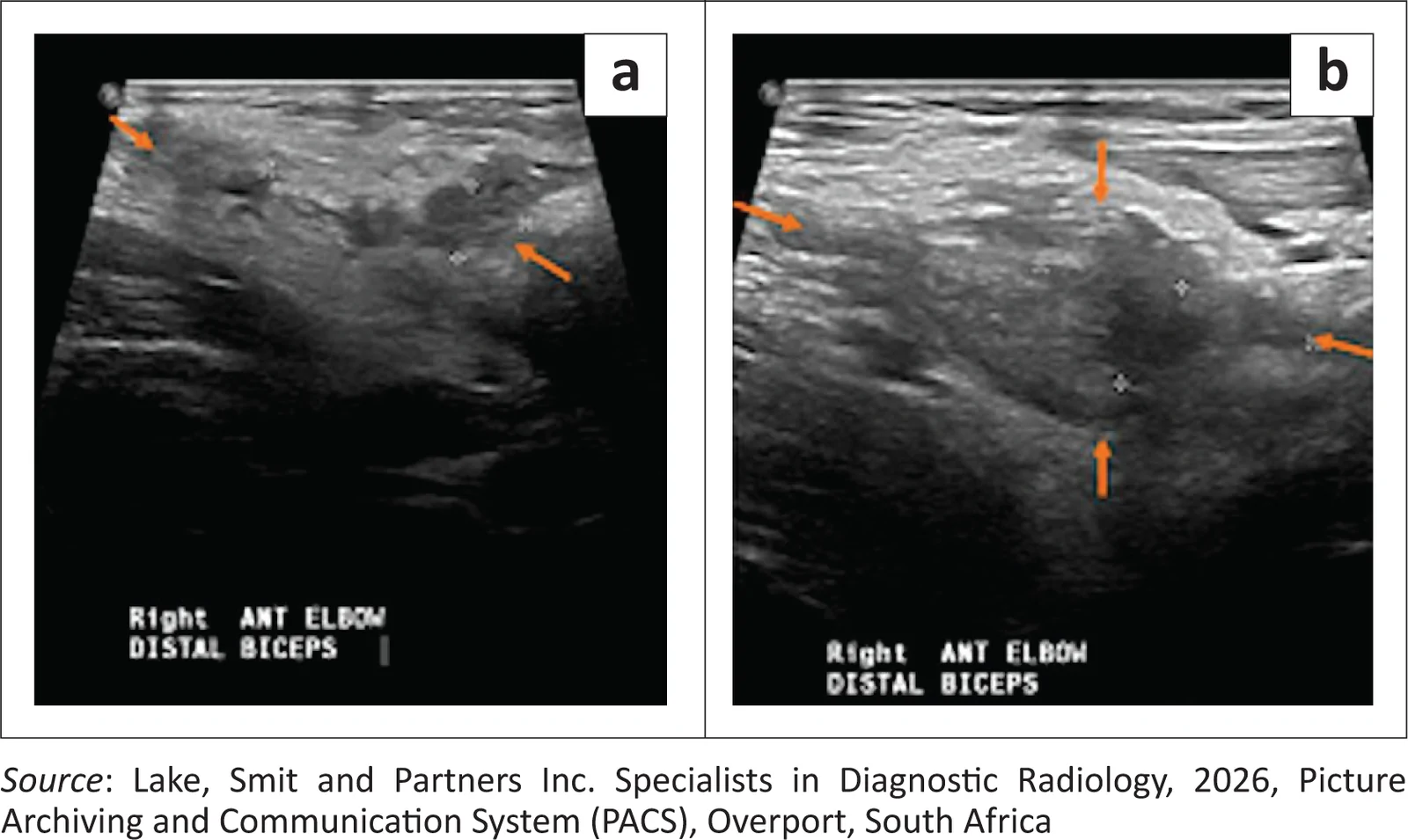
Longitudinal ultrasound images (a and b) of the anterior elbow revealing a full-thickness tear of the distal biceps insertion with an associated soft tissue haematoma.

Medial epicondylitis and posterior impingement syndrome may also be seen. MRI is the most sensitive method for detecting bone marrow oedema (BMO) and soft tissue inflammation, while USG can be used to guide procedures such as tenotomy, platelet-rich plasma (PRP) injections and needle fenestration.

The flexor tendons and the ulnar collateral ligament (UCL) are susceptible to wear and tear injuries. Excessive valgus extension overload can cause posteromedial impingement, osteophyte formation and chondromalacia of the radio-capitellar joint. Plain radiographs can help identify bony abnormalities such as osteophytes and loose bodies. MRI provides high-resolution images of the common flexor tendon and UCL tears with the highest diagnostic accuracy, assessing for concomitant osteochondral and nerve insults. Dynamic ultrasound is an emerging alternate modality to evaluate the UCL under stress.^12^

### Wrist and hand injuries

Wrist sprains are often related to quick twisting motions or falls on an outstretched hand.^10^ Due to the reduced size of the grip on a padel racquet, there is an increased amount of stress on the wrist extensors during ball impact, mostly extensor carpi ulnaris (ECU), resulting in ECU tendinopathy. Untreated ECU tendinopathy can ultimately result in ECU subluxation or rupture. MRI and ultrasound can detect ECU instability and guide treatment, ranging from immobilisation to surgical sub-sheath reconstruction. MRI can also detect associated bone contusions or fractures.^20^ Stress fractures of the metacarpals, involving the second and third metacarpals, can also be seen in padel, secondary to chronic repetitive injuries.^20^ MRI of the wrist also plays a crucial role in evaluating padel-related injuries that manifest as ulnar-sided wrist pain and helps in guiding appropriate treatment. Ulnar impaction and torsional forces at the distal radioulnar joint (DRUJ) can cause triangular fibrocartilage complex (TFCC) injuries, which are common in racquet sports. These are diagnosed on MR arthrography, and treatment includes arthroscopic debridement or repair.

Other wrist injuries encountered in padel include scapholunate ligament tears, extrinsic ligament sprains and hook of hamate fractures. MRI is the preferred modality for wrist injuries in padel, demonstrating complex central TFCC tears with fluid extending into the DRUJ (Figure 5). Plain radiographs followed by MRI are essential for detecting subtle scaphoid waist fractures and monitoring for complications such as radial osteitis in athletes who return to play prematurely (Figure 6).

**FIGURE 5:**
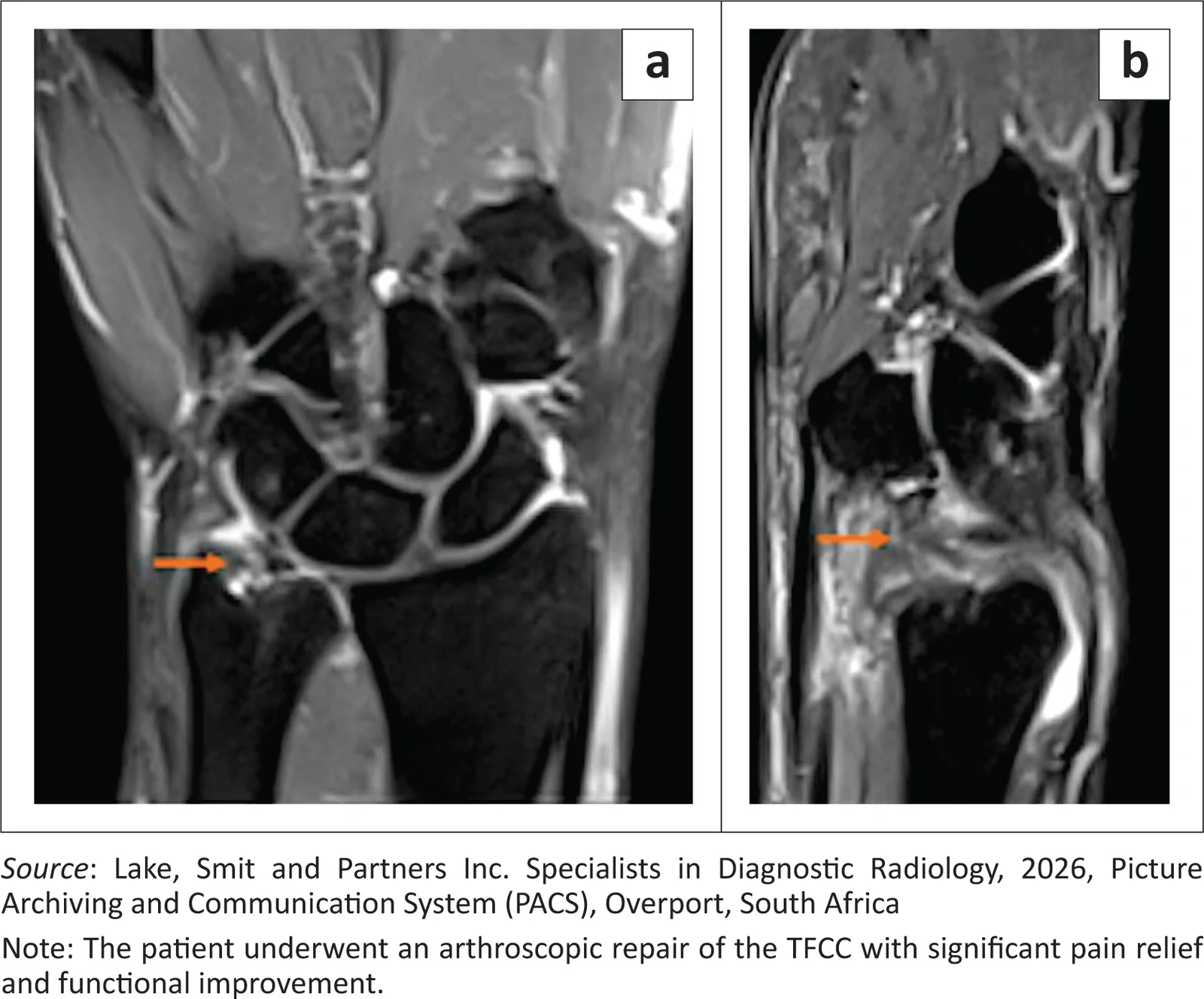
Triangular fibrocartilage complex (TFCC) tear of a 48-year-old male player who presented with wrist pain and clicking following a fall on an outstretched hand. MRI identified a complex central tear of the TFCC, with fluid extending into the distal radioulnar joint (a and b).

**FIGURE 6:**
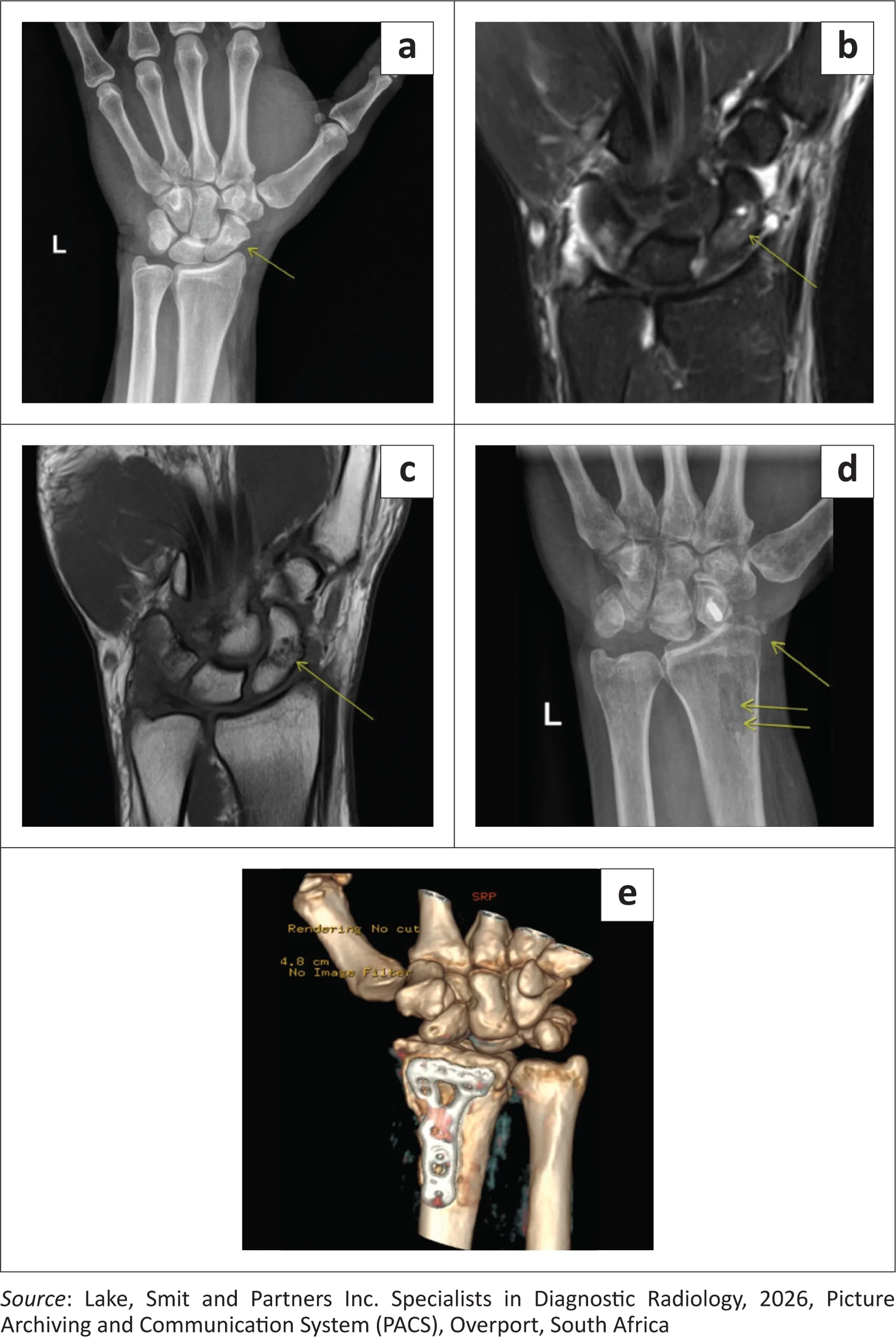
Scaphoid waist fracture and subsequent repeat injury with complicating radial osteitis in a 50-year-old male novice padel player who presented with wrist swelling and pain after a fall: The plain radiograph revealed a subtle dense line across the scaphoid waist (a), MRI demonstrated a non-displaced scaphoid fracture (b and c). The patient continued playing padel and suffered subsequent injuries of the distal radius, complicated by osteitis (d) requiring surgical fixation as displayed in (e).

## Lower extremity injuries

### Hip and Groin injuries

Hip and groin injuries are less common in padel compared to knee or elbow injuries,^14^ and include hip flexor strains, adductor strains and labral tears resulting from sudden twisting movements or repetitive stress on the hip and groin muscles.^13^ The court’s compact dimensions and surrounding walls force dynamic movements – quick directional changes, lunging and explosive rotations – placing significant shear and compressive forces on the hip joint.^20^ This can result in damage to the acetabular labrum and articular cartilage, as well as the development of cam-type femoroacetabular impingement (FAI) with associated anterior-superior labral tears. Radiographs are the first imaging study for hip and groin pain and may identify the osseous bump characteristic of cam-type FAI.

For persistent symptoms, a dedicated hip MRI is warranted to evaluate the labrum, cartilage and periarticular soft tissues, with MR arthrography being the best modality for visualising labral tears and delaminated cartilage flaps. Symptomatic FAI that fails conservative treatment may require hip arthroscopy, with preoperative MRI helping to determine the appropriate intervention. Post-operative MRI can be used to assess outcomes and rule out complications such as heterotopic ossification. Padel athletes are also prone to extra-articular groin injuries – adductor strains, sports hernias, osteitis pubis and proximal rectus femoris injuries – from repetitive pivoting and deceleration forces.

### Knee injuries

The knee is the most common lower limb region injured in padel.^12^ The common knee injuries often consist of ligament strains, meniscal tears and tendinitis.^14^ Improper playing techniques, such as foot positioning or improper body mechanics, can place extra pressure on the knees, increasing the likelihood of knee injuries.^13^

Meniscal tears are quite common, with the medial meniscus tearing twice as frequently as the lateral meniscus. MRI is usually the preferred imaging technique because it helps in visualising meniscal details and any associated injuries. While small peripheral tears may occasionally heal with conservative treatment, many athletes select arthroscopic partial meniscectomy to shorten their time to return to play.

The dreaded cruciate ligament tears also plague padel players. The anterior cruciate ligament (ACL) is more commonly injured than the posterior cruciate ligament (PCL), usually in a non-contact hyperextension pivoting injury, while the PCL is injured in direct proximal tibial impact injury. Cruciate tears are easily diagnosed on MRI, and concomitant meniscal, chondral or collateral ligament injury often coexists. MRI is the gold standard for evaluating cruciate ligament injuries, thereby guiding the decision between conservative management and surgical reconstruction (Figure 7).

**FIGURE 7:**
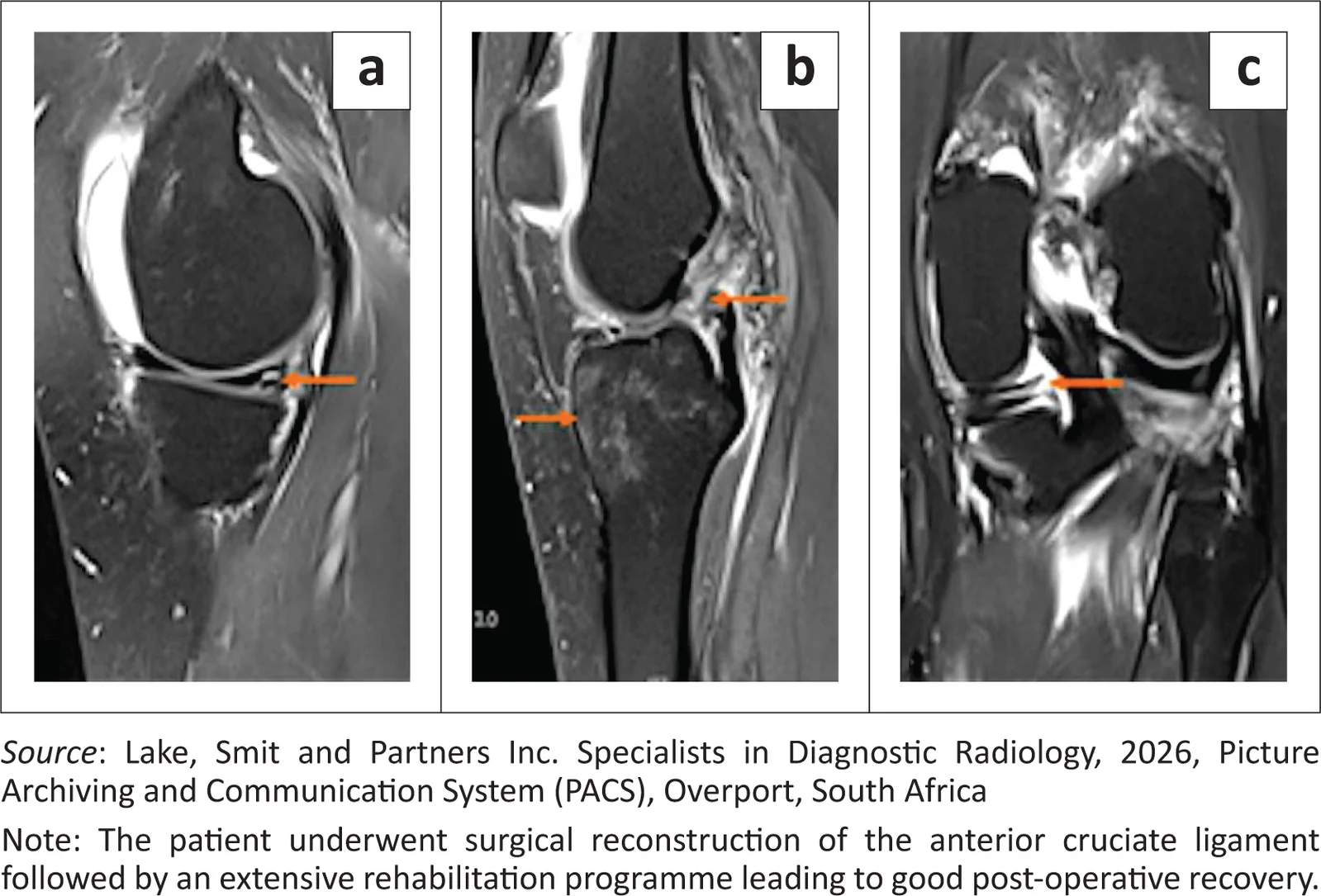
Anterior cruciate ligament and medial meniscal tear in a 37-year-old male player who presented with acute knee pain and instability after a pivot-shift pattern of knee injury. MRI revealed a complete tear of the anterior cruciate ligament with associated bone contusions and a medial meniscus tear (arrows a-c).

Reconstruction of isolated ACL tears is generally recommended to protect knee stability, while non-operative management of PCL tears is more frequently carried out. Repetitive jumping and knee flexion may also produce quadriceps tendinopathy proximally, with patellar tendinopathy occurring predominantly at the distal pole. Thickening and intrasubstance signal changes of the tendon observed on MRI and ultrasound may evolve into interstitial tearing. Ultrasound-guided treatment options, like PRP injections and percutaneous needle tenotomy, are typically reserved for cases that do not respond well to conservative treatments.

### Ankle and foot injuries

Injuries of the ankle and foot are quite common in padel due its fast-paced nature. Typical injuries involve sprains, strains and fractures often triggered by twists, awkward landings and repetitive strain during play.^14^ The lateral ankle ligament complex, which includes the anterior talofibular ligament (ATFL) calcaneofibular ligament (CFL) and posterior talofibular ligament (PTFL), is frequently affected by ankle sprains. While radiographs are usually performed to exclude a fracture, MRI is preferred for accurately assessing and grading soft tissue and ligament injuries. MRI is beneficial in athletes with residual or recurrent instabilities following prior conservative treatment and athletes who are potential candidates for surgical reconstruction. MRI of the ankle accurately delineates high-grade partial-thickness ATFL tears with surrounding soft tissue oedema and associated deltoid ligament injuries, as displayed in Figure 8.

**FIGURE 8:**
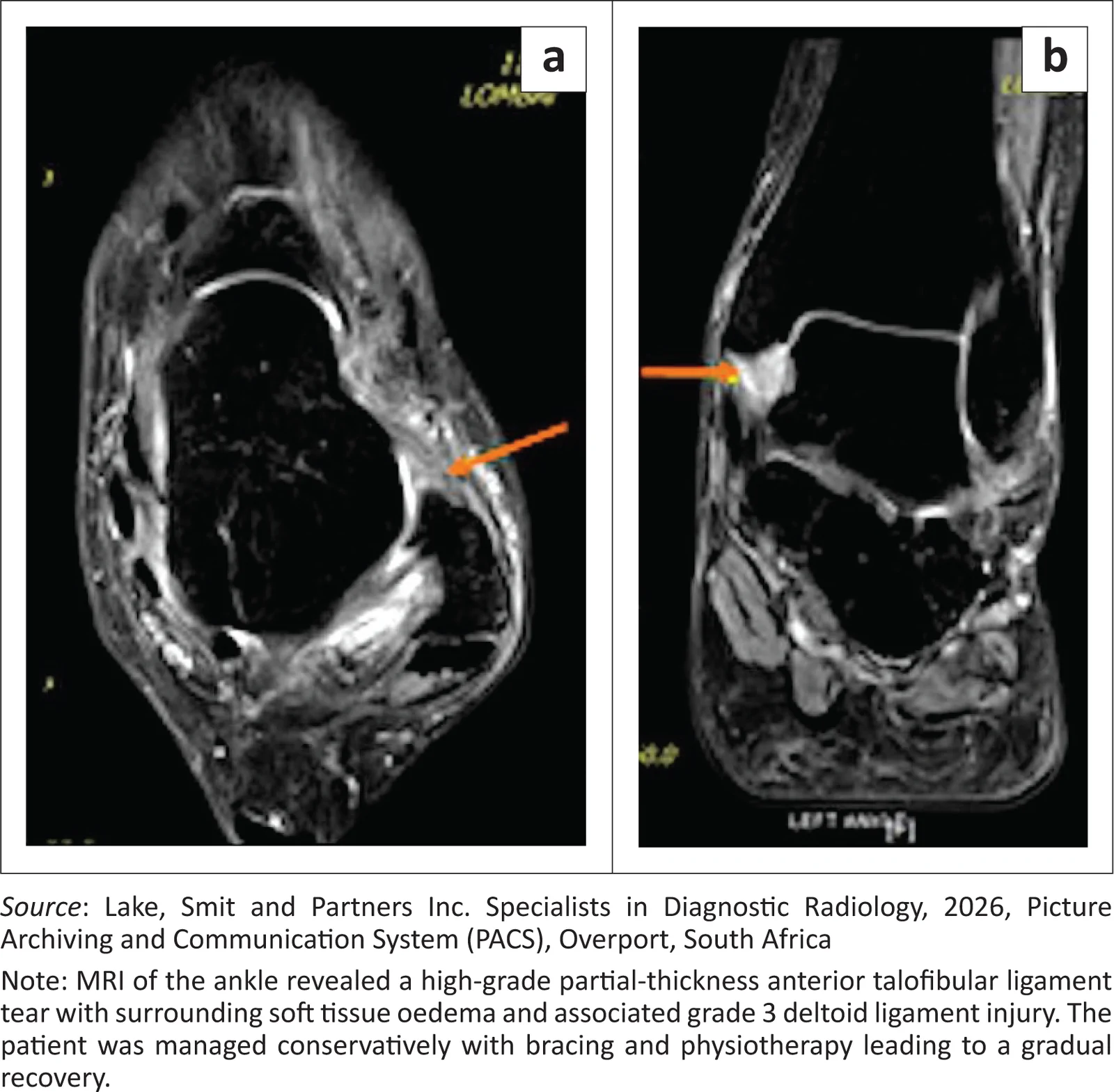
Ankle sprain of a 53-year-old male player who sustained an ankle sprain while playing padel. The mechanism was an inversion-type injury.

The up-and-down-moving, lunging and the forceful push-off generate significant mechanical load on the Achilles tendon, resulting in partial-thickness intratendinous tears, predominantly at the critical avascular mid-portion. Achilles para-tenonitis and insertional tendinopathy are also commonly seen in chronic overuse cases. Percutaneous interventions like PRP injections and needle tenotomy have been attempted to induce a healing response in non-responding Achilles tendinopathy.

A forceful push-off or pivot manoeuvre can result in an acute complete Achilles tendon rupture. Ultrasound and MRI may delineate the rupture gap and quantify the degree of tendinous degeneration and residual tendon quality to aid surgical planning and targeted load management in conservative treatment. Ultrasound is the first-line modality for calf and Achilles tendon assessment, demonstrating muscle injuries at the medial gastrocnemius–soleus junction (Figure 9), aponeurotic shearing (Figure 10), and varying degrees of Achilles tendinopathy or full-thickness tendon tears (Figure 11).

**FIGURE 9:**
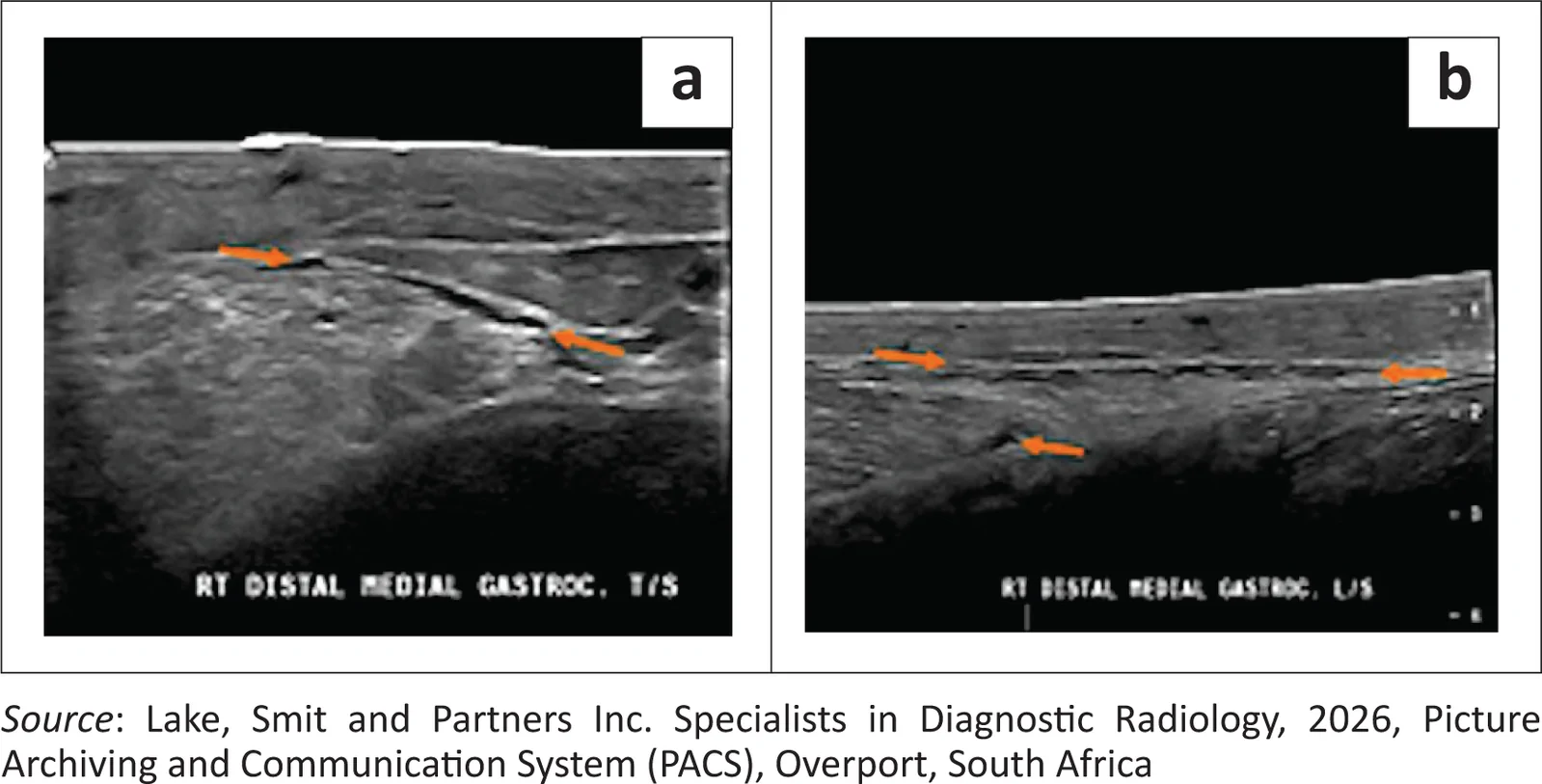
‘Tennis leg injury’: (a) Transverse and (b) Longitudinal ultrasound images of the calf revealing muscle injury at the junction of the medial gastrocnemius and soleus.

**FIGURE 10:**
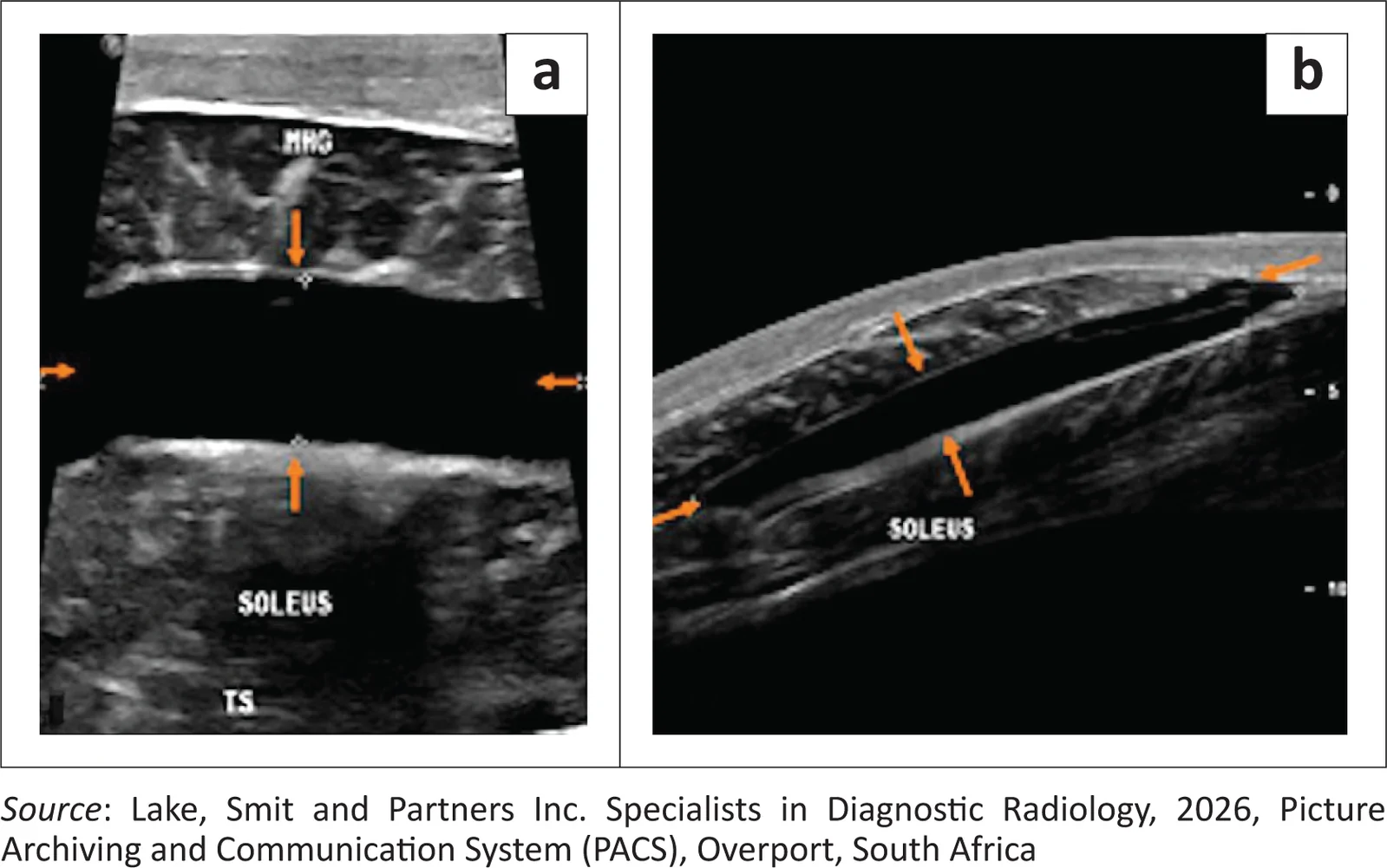
(a) Transverse and (b) Longitudinal ultrasound images of the calf demonstrating aponeurotic shearing at the junction of the medial gastrocnemius and soleus.

**FIGURE 11:**
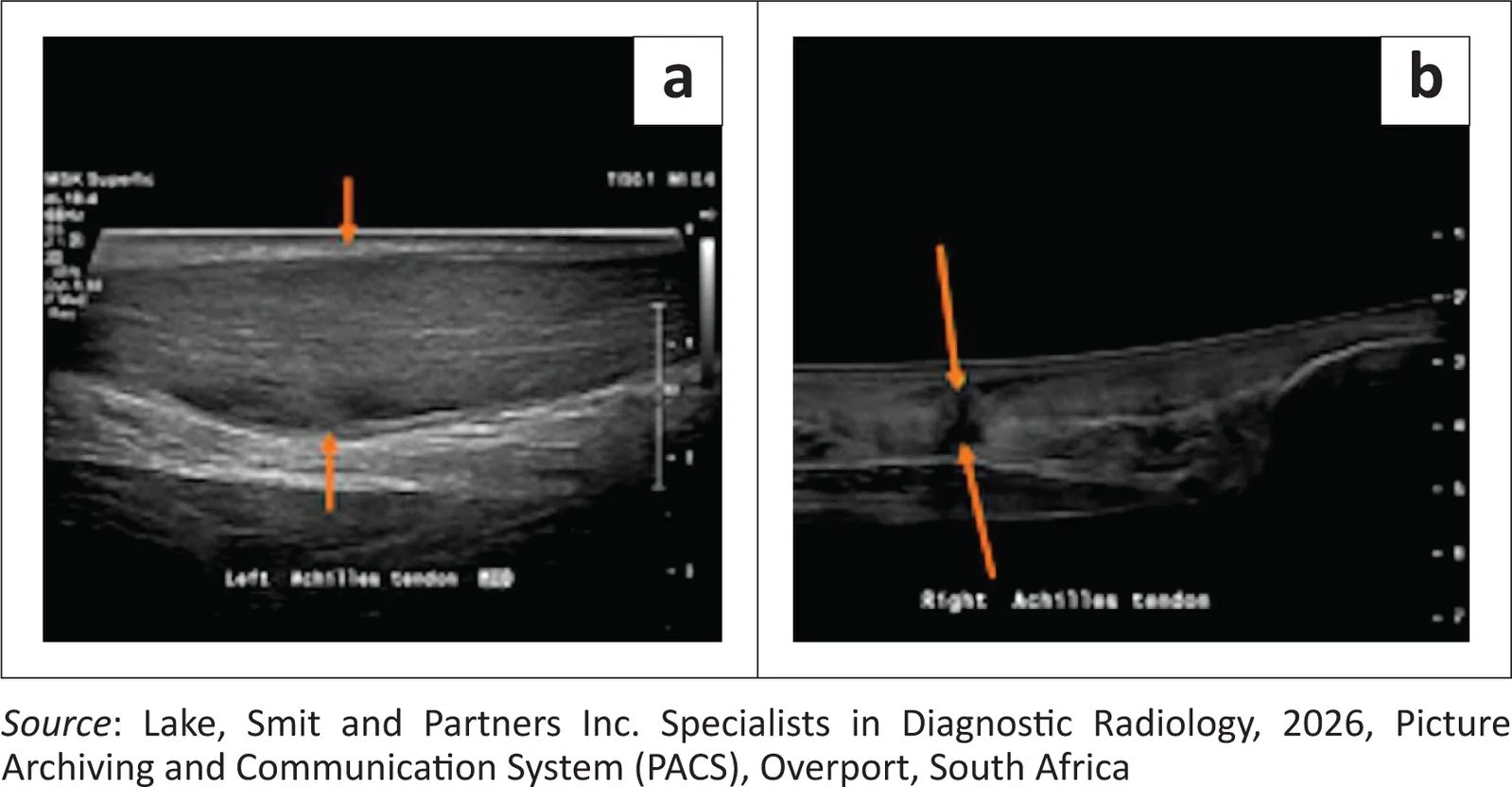
(a) Longitudinal ultrasound images demonstrating thickening of the non-insertional portion of the Achilles tendon in keeping with Achilles tendinopathy and (b) longitudinal ultrasound images showing a full-thickness tear of the non-insertional portion of the Achilles tendon.

Stress fractures are under-recognised as a source of foot pain in padel athletes. Micro-trabecular injury occurs when repetitive loads overwhelm the bone’s repair response, often due to malalignment, suboptimal equipment or rigid court surface. Metatarsal stress fractures are widespread because of the physical forces generated from being relative to rapidly undertaken lateral steps. Radiographs will be normal in the early stages of a stress fracture. MRI is the diagnostic gold standard, demonstrating a low signal fracture line with surrounding bone oedema along anatomic lines of stress. If MRI is contraindicated, bone scintigraphy or CT can be used to detect subtle fractures. Timely diagnosis and load modification can help most metatarsal stress fractures heal without surgical intervention.

## Lower back

Lower back injuries are reported less frequently than injuries to the elbow, knee or shoulder in padel.^13^ Back muscle strains, spinal ligament sprains or herniated discs are the most common reported lower back injuries, presenting with lower back pain, stiffness and difficulty in bending or twisting the back.^12^ Lower back muscle strains are typical in padel, due to the repetitive twisting and bending movements, sudden positional changes and poor posture required during play that may tear or stretch the lower back muscle fibres. Acute disc protrusion may occur due to sudden, intense movements during padel matches.

## Future directions

As padel’s popularity rises, radiologists must identify its injury patterns to improve patient care. Understanding the normal biomechanics and patho-anatomy of these injuries is paramount for early diagnosis and enhanced treatment, which in turn enables a faster return to play. If left untreated, these issues can cause persistent pain, restricted function and a prolonged absence from the sport. A multi-speciality team – radiologist, sports medicine physician and physiotherapist – provides a holistic approach to restore pre-injury performance and minimise re-injury risk. While minor sprains and strains are managed conservatively with rest, ice, compression and elevation (RICE), more significant injuries may require timely surgical intervention, such as ACL reconstruction or rotator cuff repair. Accurate preoperative MRI is essential for guiding therapeutic decisions and for post-operative assessment of healing. Injury prevention strategies, including proper warm-up and cool-down routines, strength and conditioning programmes and technique modification, are also gaining traction. As musculoskeletal radiologists, we can contribute to these strategies by identifying distinct risk factors and injury patterns on imaging.

## Conclusion

Padel is a novel racquet sport incorporating high performance and specific biomechanical properties that results in a spectrum of musculoskeletal injuries. Imaging plays a vital role in both the diagnosis and the management of these injuries. X-rays, MRI, CT and ultrasound have varying individual strengths and enable smarter imaging-based decision-making. Radiologists are central to diagnosing injury and advising subsequent management. Crucially, the MSK radiologist must understand the relevant biomechanisms, collate imaging findings and generate a timely diagnosis to facilitate clinical treatment of padel-related injuries.
